# Sources Identification and Health Risk Evaluation of 10 Heavy Metals (Metalloids) in Soils of the Aibi Lake Basin, Northwest China

**DOI:** 10.1155/2022/8201972

**Published:** 2022-05-27

**Authors:** Zhang Zhaoyong, Guo Jieyi, Wang Pengwei

**Affiliations:** ^1^School of Environment and Surveying Engineering, Suzhou University, Suzhou 2340000, China; ^2^College of Resource and Environmental Sciences, Xinjiang University, Urumqi 830046, China; ^3^Key Laboratory of Oasis Ecology, Ministry of Education, Xinjiang University, Urumqi 830046, China

## Abstract

Recently, soils heavy metals pollution and health risks researches in oasis are few, and in this study, the Aibi lake basin—a typical oasis—was chosen as the research area, and then, we evaluated the pollution status and sources identification and analyzed the health risks of ten heavy metals in the soils. Results showed that (1) the average (range) values for As, Cd, Co, Cr, Cu, Hg, Mn, Ni, Pb, and Zn were (6.500–48.040) 20.011, (0.0002–0.088) 0.035, (0.060–18.150) 5.994, (24.160–106.400) 53.557, (3.460–58.760) 16.981, (0.0002–0.099) 0.042, (195.310–842.850) 483.311, (0.960–70.100) 14.235, (0.180–25.390) 8.086, and (22.340–156.250) 61.334 mg/kg, respectively, and we can get except for As, the maximum values of other nine elements all within the limited values provided by the soil environmental quality risk control standard of China. (2) Health risk evaluation showed that the total exposure amount for ADI_ing_ for children and adults was 0.001067998 and 0.000344707, ADI_inh_ for children and adults was 9.69977E-08 and 7.95869E-08, ADI_derm_ for children and adults was 8.52275E-06 and 2.09927E-06, and the order of exploring ways is ADI_ing_ > ADI_inh_ > ADI_derm_. (3) The multivariate statistical analysis and PMF results showed that Cr, Cu, Co, Mn, Ni, Pb, and Zn primarily come from the natural background and man-made sources; Cd primarily comes from man-made sources; As and Hg come from natural background sources and industry sources. The results can provide reference values for heavy metals pollution prevention and the protection of the environment in the Aibi lake basin and as well as central Asia.

## 1. Introduction

Heavy metals are persistent toxic pollutants in the environment and have bio-accumulative and nondegradable characteristics [1–3]. Recently, the pollution of the environment by heavy metals has received the attention of many scholars worldwide. Additionally, they can directly or indirectly affect human health. Thus, research regarding the content of heavy metals in farmland soil has been widely conducted worldwide [4, 5]. The heavy metals found in soils primarily come from urban construction, urban lives, industrialization, wastewaters, dust, and solid wastes from smelt plants, and oil and mining explorations and exceed the amount of heavy metals present from the use of pesticide chemical fertilizers [6, 7].

Previous studies have focused on the spatial distribution, source identification [8], pollution assessment, evaluation of the health risks [9], and environmental risks [10]. Methods used include GIS technology, the enrichment factor method [11], the geo-accumulation index [12], the Hakanson potential risks index [9], the health risks evaluation model [13], multivariate statistical analysis, positive matrix factorization (PMF) [14], and risk assessment coding (RAC) [10].

Initial health risk models by USEPA were carried out using soils, dust, waterbody, and the fruit of plants and evaluated the carcinogenic and noncarcinogenic risks of heavy metals [2, 15]. There are now relevant researches about soil heavy metals health risks evaluation in the world, such as in the study by Li et al. [16], a human risk assessment was carried out for heavy metals in the abandoned metal mine areas of Korea, and the calculated hazard index value for As in the Songchun mine area (3.625) exceeded 1.0. In a study by Harmanescu et al. [17], they carried out a heavy metal health risk assessment for a population via the consumption of vegetables grown in an old mining area in Romania. Al-Hwaiti and Al-Khashman [9] carried out a health risk assessment of heavy metal contamination for the following metals: Cd, Cr, Cu, Pb, V, and Zn in tomato and green pepper plants grown in soils amended with phosphogypsum (PG) waste materials and found that the daily intake of metals (DIM) and the health risk index (HRI) values were <1. According to the first national soil pollution survey of China in 2014, the soil in China currently faces a serious threat from heavy metals pollution [18]. The survey showed that the point exceeding the rate of soil in China was 19.4%, with a slight proportion, mild, moderate, and severe pollution points of 13, 7%, 2.8%, 1.8%, and 1.1%, respectively. The majority of the polluted elements were identified to be Cd, Ni, Cu, As, Hg, and Pb [18].

Nowadays, the report has revealed that 64.8% of the 140 km^2^ of the wastewater irrigation regions in China were shown to be polluted with heavy metals, resulting in an overall reduction in crop production reaching 10 million tons [19]. Such as Luo et al. [10] estimated the input/output fluxes of heavy metals in Chinese soil and showed that the input fluxes of heavy metals in most farmland were about 3–140 times the output fluxes. The annual input flux of Cd in farmland soil has been reported to be as high as 1417t [13]. Nowadays, researches regarding heavy metals soil pollution are widely conducted throughout China, such as Shenyang city, Liaoning province, the Pearl River Estuary, the Guangdong province, Wuxi city, and the Yangzhou district in the Jiangsu province [20] while these studies have primarily focused on eastern China. However, little research has been done on oasis basins, especially those located in the arid regions of China.

In this study, the Aibi lake basin, a typical oasis in northwest China and Central Asia, was chosen as the research area. We first sampled soils in the whole basin, and after test of ten metals As, Cd, Co, Cr, Cu, Hg, Mn, Ni, Pb, and Zn, a variety of methods were carried out including the enrichment factor method, the PMF and health risk models from United States Environmental Protection Agency (USEPA), and the multivariate statistical analysis method to reveal the sources, distribution characteristics, and health risks of ten heavy metals in soils of the Aibi lake basin. The results can be used as references for the heavy metals pollution prevention, as well as human health protection for the arid oasis in northwest China.

## 2. Materials and Methods

### 2.1. Research Area and Sampling Sites

**Figure 1:**
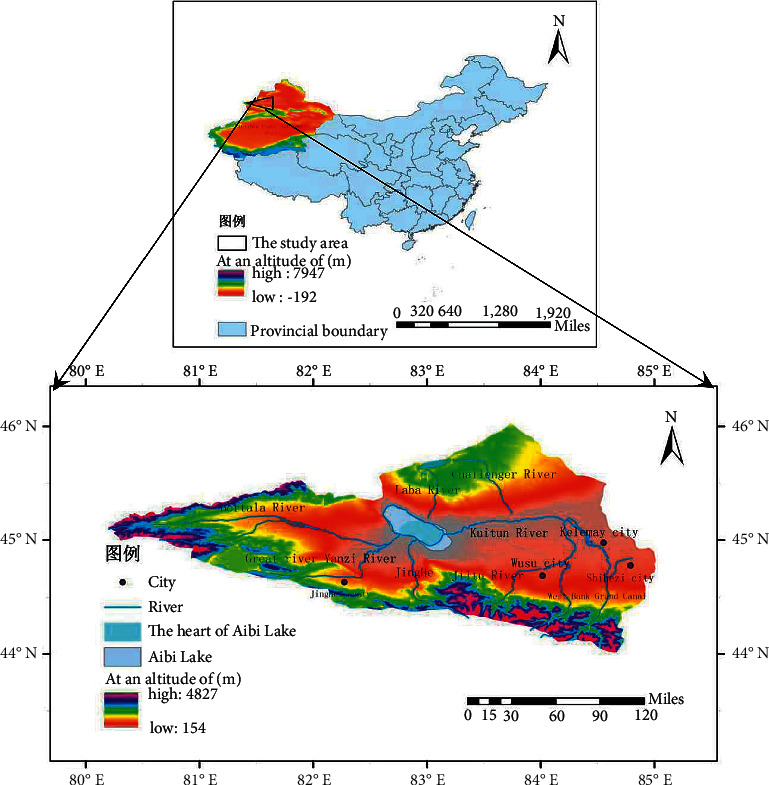
Map of the study area.

The Aibi lake basin is located in the western Xinjiang arid region of China, with an altitude between 43°38'–45°52′N and a latitude between 79°53′–85°02′E (Figure 1). Aibi lake is the largest saltwater lake in Xinjiang and the Junggar Basin. The Aibi lake basin has a dry climate with little precipitation. The average annual temperature is 8.3 °C, and the average annual precipitation is 90.9 mm. The average annual precipitation on the surface of the lake is about 95 mm, and the annual evaporation can reach as high as 1315 mm [21]. The flora of the Aibi lake basin is influenced by the flora of central Asia and Mongolia, and there are 385 types of plants belonging to 191 different genera, encompassing 53 families [12]. In the west, it is located in the main passage area of strong winds at the mouth of the Alaskan mountain, and strong winds (category 8) with a maximum wind speed of 55 m/s are more likely occurred from April to June [21].

**Figure 2:**
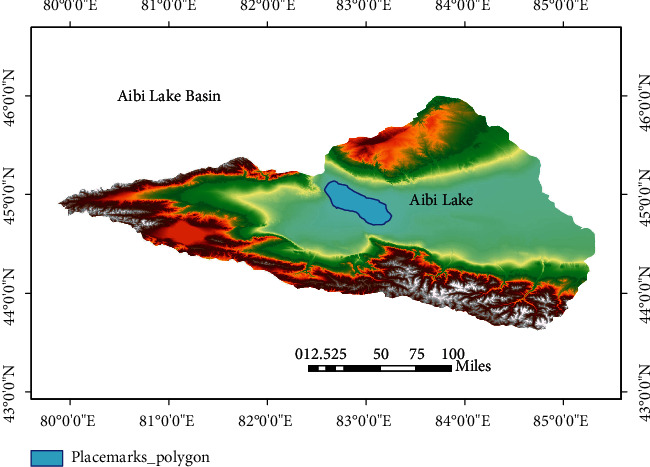
Map of the sampling sites.

Soil samples were collected from the whole Aibi lake basin from August to September of 2018. During the process, a combination of the grid method and 3S technology was used, and eventually, we get a total of 550 soil points, and the sampling interval was 1.5 km × 1.5 km (Figure 2). All soil samples were collected from 0 to 10 cm of the research area, and 400 g samples were collected at each point, and then, they were stored in polyethylene sample bags. During the sampling process, the numbers, sampling locations, sampling dates, and notes regarding the surrounding environment were recorded for further analysis.

In the laboratory, each soil sample was dried at room temperature, to removal of plant debris and rocks, then through a 100-mesh sieve and stored. The determination of 13 heavy metals was carried out as follows: first, weigh 0.2 g of the soil sample and place it in the Anton PVC digestion tank. After digestion, seal it in the digestion apparatus, heat it up to 170 °C for 30 min, cool it, then remove the sample, and collect them, to determine the volume. The contents of 13 heavy metal elements (Al, As, Cd, Co, Cr, Cu, Fe, Hg, Mn, Ni, Pb, Sc, and Zn) were determined using an inductively coupled plasma mass spectrometer (ICP-MS, Agilent 7700). The detection limits of the test instrument for the elements are all lower than 0.01 mg/kg. The accuracy and precision of the analysis method were tested using national level 1 soil reference material (GBW series), and the recovery of various metal elements was within the allowable range of the national standard reference material.

The determination of lead isotope was carried out as follows: the powder was completely dissolved in HF-HNO_3_ to mix acid at a high temperature and then extracted with 0.6 M HBr acid. The lead samples were separated and purified on a Teflon exchange column with 150 *μ*L AGIx8 (100–200 mesh) exchange resin with 0.6 M HBr and 6 M HCl acid. The isotope ratios of lead and copper were determined by MIC-ICP-MS (Neptune plus, Sommerfeld company, Germany).

### 2.2. Enrichment Factor Method

Nowadays, the enrichment factor method is widely used to determine the pollution status and sources of heavy metals in the environment. The format is as follows [5, 22]:

(1) $$\begin{array}{c} \text{EF}=\frac{{\left(C_{x}/C_{\text{ref}}\right)}_{\text{sample}}}{{\left(C_{x}/C_{\text{ref}}\right)}_{\text{background}}}, \end{array}$$

where EF is the enrichment factor of a certain heavy metal, *C*_*x*_ is the tested concentration (mg·kg^−1^), and C_ref_ is the concentration of a reference element (mg·kg^−1^). In this study, the contents of Al, Fe, and Sc in the soil of the Aibi lake basin were used as reference elements. *C*_*x*_/*C*_ref_ is the ratio of the concentration of a specific element to the concentration of a reference element.

The background values for 10 elements are used as the background value for Xinjiang (CNEMC, 1990). The values of EFs can typically be classified into five grades: EF < 2, indicating no pollution (<1) and slight pollution (1–2); 2 < EF < 5, indicating a moderate pollution level; 5 < EF < 20, indicating a significant pollution level; 20 < EF < 40, indicating a strong pollution level; 40 < EF, indicating an extreme pollution level [23].

### 2.3. PMF Method

Positive-definite matrix factorization (PMF) is an ideal receptor model for source apportionment, which is recommended by the USEPA for source apportionment based on species composition data set [24]. PMF model can decompose the matrix of the original data set and decompose X_ij_ into two factor matrices: source contribution matrix g_ik_ and source configuration matrix f_ik_. The basic equation is as follows:

(2) $$\begin{array}{c} X_{ij}=\sum_{k=1}^{0}g_{ik}f_{jk}+e_{ij}, \end{array}$$

in which, the input data set can be regarded as a *X*_*ij*_ matrix, where *X*_*ij*_ represents the concentration of the heavy metal *j* at the sampling point *i*, *g*_*ik*_ represents the contribution of the pollution source *k* in the sample *i*, *g*_*ik*_ represents the concentration of element *j* from the source *k*, and *e*_*ij*_ residual error matrix can be calculated by the minimum value of objective function *Q*. The value of *Q* is calculated as follows:

(3) $$\begin{array}{c} Q=\sum_{i=1}^{n}\sum_{j=1}^{m}{\left(\frac{e_{ij}}{u_{ij}}\right)}^{2}, \end{array}$$

where *U*_*ij*_ is the uncertainty of the heavy metal *j* in the sample *i*, and there are many methods to calculate the uncertainty. In this research, they were input into EPA PMF5.0 software for analysis and identification. In this research, the ROBUST mode is adopted, the number of runs is set to 20, and the model was run 20 times [22].

### 2.4. Health Risk Evaluation

#### 2.4.1. Calculation of the Exposure Quantity

In this study, the format is referenced from the United States Environmental Protection Agency (USEPA). We calculated the exposed quantity of health risks through hand-mouth intake, respiration intake, and skin exposure [4, 24].

(4) $$\begin{array}{c} {\text{ADI}}_{\text{ing}}=\frac{95\%UGL\cdot R_{\text{ing}}CF\cdot EF\cdot E\;D}{BW\cdot AT}, \end{array}$$

(5) $$\begin{array}{c} {\text{ADI}}_{\text{inh}}=\frac{95\%UGL\cdot R_{\text{inh}}\cdot EF\cdot E\;D}{PEF\cdot BW\cdot AT}, \end{array}$$

(6) $$\begin{array}{c} {\text{ADI}}_{\text{dersm}}=\frac{95\%UGL\cdot SA\cdot \cdot SL\cdot ABS\cdot EF\cdot E\;D\cdot CF}{BW\cdot AT}, \end{array}$$

(7) $$\begin{array}{c} {\text{LADD}}_{\text{inh}}=\frac{C\cdot EF}{PEF\cdot AT}\cdot \left(\frac{R_{\text{inh child}}}{BW_{\text{child}}}\cdot ED_{\text{child}}+\frac{R_{\text{inh adult}}}{BW_{\text{adult}}}\cdot ED_{\text{adult}}\right), \end{array}$$

where ADI_ing_ is the average amount of daily exposure through hand-mouth intake (mg·(kg·d)^−1^); ADD_inh_ is the average amount of daily exposure through respiration (mg·(kg·d)^−1^); ADI_derm_ is the average amount of daily exposure through skin exposure (mg·(kg·d)^−1^); LADD_inh_ is the average amount of daily exposure for life of carcinogenic heavy metals through respiration (mg/(kg·d)); EF is the frequency of exposure to the human body, which was chosen to be 250 day·a^−1^; ED is the fixed number of years exposed, which was set at 6 yrs and 30 yrs for children and adults, respectively [25]; AT is the average exposure time, which was chosen to be 365 × ED for noncarcinogenic heavy metals and 365 × 70 for carcinogenic of both children and adults [26]; BW is the average weight of the human body, which was set at 15 kg and 60.6 kg for children and adults, respectively [25, 27]; CF represents the unit converter, which was 1 × 10^−6^ for both [28]; R_ing_ is the consumption rate of soil by way of hand-to-mouth, which was chosen to be 200 and 50 mg kg^−1^ for children and adults, respectively; R_inh_ is the respiratory rate of the human body, which was chosen to be 5 and 15.7 m^3^·day^−1^, respectively [25, 27]; PEF represents the particulate emission factor of heavy metals, which was 1.36 × 10^9^ m^3^ kg^−1^ [28]; SL is the skin adhesion degree, which was 0.2 and 0.07 mg cm^−1^, respectively [28]; SA is the area of exposed skin, which was 1600 cm^2^·day^−1^ and 4350 cm^2^·day^−1^, respectively [25]; ABS is the skin factor, which was chosen as 0.001 for both children and adults [20].

#### 2.4.2. Health Risk Representation

*(1) Noncarcinogenic risks*. According to the USEPA, the noncarcinogenic risk of ten (metalloid) heavy metals was calculated based on the following formula [29, 30]:

(8) $$\begin{array}{c} HQ_{ij}=\frac{{\text{ADI}}_{ij}}{{\text{RfD}}_{ij}}. \end{array}$$

The total value of the noncarcinogenic risk of ten (metalloid) heavy metals was calculated based on the following formula (HI) [29, 31]:

(9) $$\begin{array}{c} HI=\sum HQ_{ij}=\sum \frac{{\text{ADI}}_{ij}}{{\text{RfD}}_{ij}}, \end{array}$$

where HQ_ij_ represents the noncarcinogenic risk; ADI_*ij*_ represents the daily exposure dose of heavy metals through three ways (mg·(kg·d)^−1^); RfD_*ij*_ is the reference dose (mg·(kg·d)); *i* represents a certain heavy metal; *j* represents a certain route of exposure (Table 1).

*(2) Carcinogenic risk assessment*. In the research, As, Cr, Ni, Cd, and Co have the potential to pose a carcinogenic risk [31, 32]. The carcinogenic risk of these through respiratory exposure was calculated based on the following formula [33, 34]:

(10) $$\begin{array}{c} \text{Risk}=\sum \frac{LA\;DD_{inh}}{SF},\\ \text{Risks}=\sum \text{Risk}, \end{array}$$

where Risks represents the possibility of people getting cancer; *LADD*_inh_ is the exposure amount of heavy metal with a carcinogenic risk (mg·(kg·d)^−1^); *SF* is the carcinogenic slope factor (mg·(kg·d))^−1^. In the research, the carcinogenic risk through respiratory exposure parameters for five (metalloid) heavy metals is Cd-6.4, As-0.0043, Ni-0.84, Cr-42, and Co-9.8 [6, 35].

## 3. Results and Analysis

### 3.1. Statistical Characteristics of the Heavy Metals in the Soils of the Aibi Lake Basin

**Figure 3:**
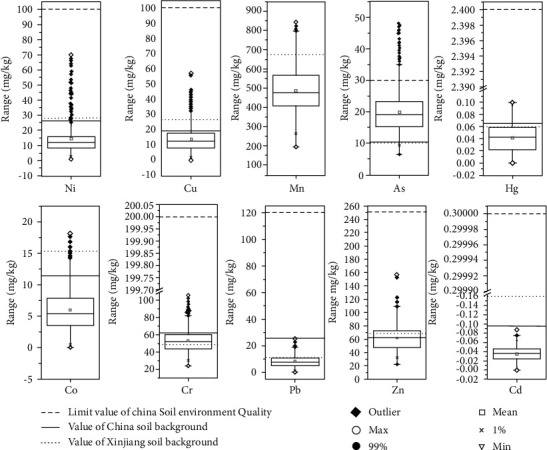
Statistical characteristics of the ten elements in the soils of the Aibi lake basin.

Statistical analyses showed that the average (range) values for As, Cd, Co, Cr, Cu, Hg, Mn, Ni, Pb, and Zn were (6.500–48.040) 20.011, (0.0002–0.088) 0.035, (0.060–18.150) 5.994, (24.160–106.400) 53.557, (3.460–58.760) 16.981, (0.0002–0.099) 0.042, (195.310–842.850) 483.311, (0.960–70.100) 14.235, (0.180–25.390) 8.086, and (22.340–156.250) 61.334 mg/kg, respectively (Figure 3). The coefficient of variation (CV) for As, Cd, Co, Cr, Cu, Hg, Mn, Ni, Pb, and Zn was determined to be 12.591%, 0.027%, 6.277%, 24.413%, 15.362%, 0.044%, 240.549%, 20.449%, 7.327%, and 33.717% [31].

The results showed that Mn had high variation, Cd, Co, Hg, and Pb had small variation, and the other five elements had moderate variation [12]. The skewness values were calculated for all ten elements, which were ordered from highest to lowest skewness value as Ni > Cu > As > Cr > Co > Zn > Pb > Mn > Hg > Cd. Nine of the elements were shown to have a positive skewness value, with Cd being the only element that had a negative skewness value. Elements with a negative kurtosis value include Cd, Mn, and Hg while the other five elements had a positive kurtosis value.

Compared with the limited available values for soil in the environmental quality risk control standard for soil contamination of agricultural land of China (6.5<pH < 7.5) [36], the exceeding rate of As in all soil samples was 7.143%, while the exceeding rate for the other seven elements was 0. Compared with the background values reported for soils in China [37], the exceeding rate of the other eight elements was determined to be As (94.548%), Co (5.525%), Cr (22.932%), Cu (20.037%), Hg (19.048%), Mn (20.404%), Ni (7.909%), and Zn (21.691%). These results are consistent with the high background values reported for As in the soils of the Aibi lake basin. Compared with the reported background values in the soils of Xinjiang [19], the exceeding rate of nine elements was determined to be As (96.053%), Cd (0%), Cr (65.79%), Cu (8.534%), Hg (23.626%), Mn (7.537%), Ni (6.97%), Pb (21.179%), and Zn (50%).

### 3.2. Enrichment Factor

**Figure 4:**
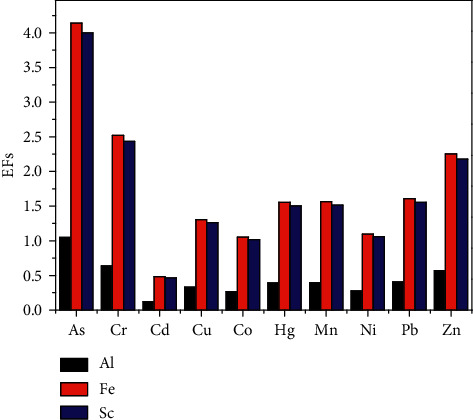
EF values of the heavy metals in the soils of the Aibi lake basin.

Using Al, Fe, and Sc as reference elements, we calculated the EF values for the ten elements. Our results showed that (Figure 4) when Al was set as the reference element, As belongs to the slight level, while the EF values for the other nine elements were all at levels considered to be no pollution. On the whole, the ten elements arranged in the order of their pollution status are as follows: As > Cr > Zn > Pb > Mn > Hg > Cu > Ni > Co > Cd.

When Fe was used as the reference element, As, Zn, and Cr belonged to a moderate pollution status, and Cd in the soils belonged to no pollution level, while Co, Cu, Hg, Mn, Ni, and Pb belonged to a slight pollution level. On the whole, the order of pollution levels of ten elements is As > Cr > Zn > Pb > Mn > Hg > Cu > Ni > Co > Cd.

When Sc was used as the reference element, As and Cr belonged to moderate pollution level, and Cd belonged to no pollution level, while Co, Cu, Hg, Mn, Ni, Pb, and Zn belonged to slight pollution levels. Eventually, the order of pollution levels of them is Cr > Zn > Pb > Mn > Hg > Cu > Ni > Co > Cd. In the study, Al, Fe, and Sc were chosen as reference elements. The results of Fe and Sc are consistent.

### 3.3. Health Risks of Soil Heavy Metals in the Aibi Lake Basin

Calculations of the exposure amount of (metalloid) heavy metals in soils showed that the total exposure amount of ADI_ing_ for children and adults was 0.001067998 and 0.000344707, respectively. ADI_inh_ for children and adults was 9.69977E-08 and 7.95869E-08, respectively, and ADI_derm_ for children and adults was 8.52275E-06 and 2.09927E-06, respectively (Table 2). The analyses showed that with the exception of Mn, the ADI_ing_ values for the other nine heavy metals were all higher for children than for adults, and with the exception of Cr and Mn, the ADI_ing_ values for eight metals were all over one order of magnitude greater for children than for adults. The ADI_inh_ values of As, Cd, Co Cr, and Ni were observed to be higher for adults than for children, and the ADI_inh_ values of Cu, Hg, Mn, Pb, and Zn were observed to be higher for children than for adults. The ADI_derm_ values of Co, Cu, Pb, Hg, and Mn were higher for children than for adults, and the values of As, Cr, Co, Ni, and Zn were higher for adults than for children.

When the HQ or HI value was <1.0, the noncarcinogenic risk was recognized as being relatively low and safely ignored. When the HQ or HI value was ≥1.0, health risks were considered to be present. Calculations of the noncarcinogenic risk (HQ) of the ten (metalloid) heavy metals showed that HQ_ing_, HQ_inh_, and HQ_derm_ for both children and adults were all less than 1, and the HI of the three HQs for children and adults was also less than 1, indicating that there was no noncarcinogenic risk for the ten elements (Table 3). Among the ten elements, the HQ for Pb for children reached the maximum value of 0.021097286, and the second highest was HQ_inh_ for As for adults at 0.016155. Considering both children and adults, the order of noncarcinogenic risks for the three routes of exposure for the ten elements is HQing > HQderm > HQinh.

The calculation of the carcinogenic risks showed that the values of As, Cd, Co, Cr, and Ni were 4.1299E-09, 1.56155E-11, 6.0499E-12, 1.58151E-07, and 8.40675E-10, respectively. The total risks for the five heavy metals were 1.63143E-07. Within the scope of the limited reference values of 10^−6^-10^−4^ by the USEPA and the International Commission on Radiation Protection (ICRP) maximum acceptable risk value of 5.0E-5 as the basis for our health risk discrimination, our results demonstrated no carcinogenic risks for the heavy metals tested [34].

### 3.4. Sources Identification of 10 Heavy Metals (Metalloid) in Soils of the Aibi Lake Basin

**Figure 5:**
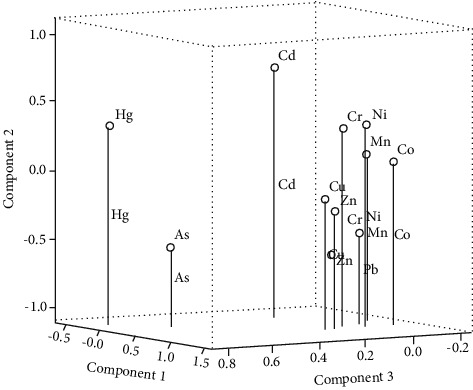
Principal components of the heavy metals in soils.

The results of the principal component analysis method showed that ten elements all fall into three principal components (Figure 5). The first component explained 48.34% of the total sources of the ten elements, the second component explained 15.044%, the third component explained 9.838%, and the cumulative explanation explained 73.221%, indicating an effective analysis. The first component contained Cr, Co, Cu, Mn, Ni, Pb, and Zn; the second component contained Cd; and the third component contained As and Hg.

The Pearson correction analysis method was used to reveal the corrections among the ten elements. The result showed that Mn-Ni, Mn-Pb, Mn-Zn, Ni-Pb, Ni-Zn, and Pb-Zn are strongly corrected at *P* < 0.01 as 0.665, 0.448, 0.669, 0.221, 0.459, and 0.663, respectively. The correction coefficient for Cr-Cu was 0.583, which is significant at *P* < 0.01. Except for Co-As and Co-Hg, there was a significant correction coefficient between Co and the other seven elements at *P* < 0.01 or 0.05. Cr and Cu were also shown to have significant correlations with Co, Mn, Ni, Pb, and Zn (Table 4), indicating that they have the same source or an influential factor, which is in agreement with the results of the principal component analysis.

Ni-Hg and Ni-As were shown to have negative correction coefficients, −0.066 and −0.023, indicating that they may be influenced by two different factors, which is the same for Cr-As as 0.088 at *P* < 0.01. The correction coefficients of Cr-Hg, As-Hg, and Cu-Hg were −0.067, −0.03, and −0.075, respectively, indicating different influential factors. Hg-Co was significant at *P* < 0.01, indicating that they have different sources. Our analysis also showed that the correction coefficients of Hg-Pb and Hg-Zn at *P* < 0.01 were −0.448 and −0.669, respectively. Hg−Mn at *P* < 0.05 level was −0.089, and the correction coefficient of Hg-Ni was −0.066, indicating that they had different sources. The man-made sources of Hg could then be revealed by combining it with the background of soils in the Abi lake basin. As and Co primarily come from natural backgrounds.

The conduct of the PMF model showed that the lower Q value is 91.4, and all residual values are between -2 and 2. The calculation results tend to be stable. Through the positive matrix factor analysis model, the fitting results between the measured content value and the predicted value of the model are greater than 0.75, indicating that the overall analysis effect of the model is good, and the selected number of factors can fully explain the information contained in the original data and meet the needs of source analysis.

**Figure 6:**
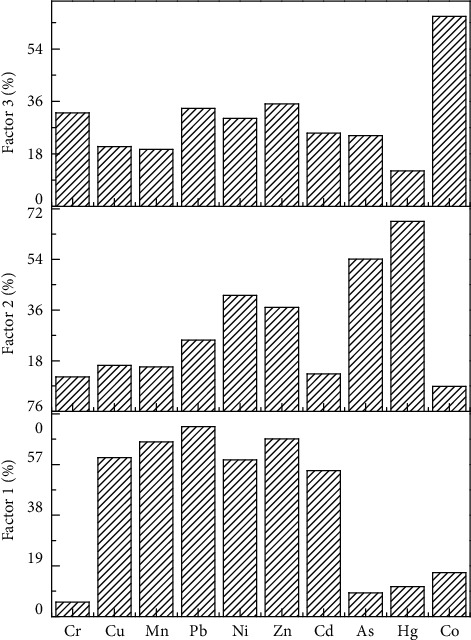
Analytical contributions of soil heavy metals PMF sources.

According to the analytical results of the positive definite matrix factor analysis model, the relative contribution rate of each source factor to each heavy metal is shown in Figure 6. From the source analysis results, it can be seen that the relative contributions of factors 1 to Co, Cr, Cu, Mn, Ni, Pb, and Zn are high. From the analysis, we can get Cr and Cu may come from the exhaust emissions of gasoline and diesel vehicles, Ni is the characteristic element of fuel combustion, Zn is the characteristic element of rubber tire and brake wear and exhaust emission, and Pb is related to motor vehicle exhaust emission and rubber tire wear. Therefore, factor members can be used as the source representatives of road mobile sources [22, 38]. Factor 2 is that Cd should mainly come from the use of Cd-containing herbicides in agricultural production [39, 40]. Factor 3 is As and Hg, mainly from the natural geographical background, which is related to the high background values in the soil environment in this area [1], and As and Hg are also influenced by emissions of waste gases and wastes containing Mercury and arsenic from coal-fired enterprises, power plants, and factories in winter [12].

### 3.5. Pollution Statue and Health Risks of 10 (Metalloid) Heavy Metals in Soils of the Aibi Lake Basin

Compared with the results in previous studies (Table 5), we can get that the contents of heavy metals in the soils of the Aibi lake basin are lower than that in the soils in Xuzhou, China [42], the soils of Tehran, Iran [43], the dust in Selangor, Malaysia [5], the dust in 72 examined mine in the eastern part of China [34], as well as the soils in 146 cities of China [5]. But the contents of heavy metals in the soils of the Aibi lake basin are higher than that of the dust in Zhundong and Urumqi, Xinjiang, China [41].

The health risk assessment showed that the exposed ways of ten heavy metals both for children and adults are all in the following order: ADI_ing_ > ADI_inh_ > ADI_derm_. For both ADI_ing_ and ADI_derm_, they are higher for children than for adults, while for ADI_inh_, they are higher for adults than for children. These results are in agreement with the research of soil heavy metals in arid regions such as in soils along the Central Elbe River, Germany [44], Urumqi [31], Xiong'an [15], and Shenzhen [2] of China, while they are different from the research results obtained from Lanzhou (ADI_ing_ > ADI_derm_ > ADI_inh_), which has a very serious pollution situation of heavy metals [34]. In noncarcinogenic risks calculation, when HQ or HI value is < 1.0, the noncarcinogenic risk is recognized as being relatively low and can be ignored. The noncarcinogenic risk calculation results of the current study are consistent with those in the research of Istanbul, Turkey [43], Shanghai [45], Xi'an [6], and also in Zhundong, Chanji city, Xinjiang in arid regions of the Xinjiang, China [41]. For carcinogenic risks analysis, in the research for five elements, Cr has the maximum risk, followed by Co and As, consistent with prior research in Baiyin [46], Gansu province, Xiong'an, Hebei province [15], and also Urumqi [41], Xinjiang, China, indicating that there is no carcinogenic risk of soil heavy metals in these areas.

During health risk evaluation, the coefficients used in the models mainly come from the standards of China as Technical Guidelines for Risk Assessment of Contaminated Sites (HJ 25.3–2014) from the Ministry of Environmental Protection of China (BW, R_ing_, and R_inh_) [47] and Environmental Site Assessment Guide (DB11T-656--2009) of Environmental Protection Bureau of Beijing, China (ABS, EF, ED, BW, R_ing_, R_inh_, and SA) [20, 25]. Yang et al. [41] found that the respiration rates of Americans (males and females) were higher than those of Chinese. The respiration rates of Chinese male children were approximately 9.0–41.3% more than American male children, while adult Chinese males had approximately 10.0–32.5% lower respiration rates than adult American males. Therefore, in future research, development and application of human health risk assessment coefficients suitable for the Chinese are the focus of efforts.

## 4. Conclusions

The following conclusions can be drawn from the study:

1. With the exception of Cd and Pb, the other eight heavy metals all exceeded the Chinese background values, among which As was shown to exceed the background value the most (94.548%). Additionally, except Co (which has no standard value), nine elements were shown to exceed the background values of Xinjiang, with the following elements showing the greatest increase above background values: As (96.053%), Cd (65.79%), Zn (50%).
2. Health risk evaluation showed that the three exposed ways for children were all higher than for adults, and the order of noncarcinogenic risks for the three routes of exposure for the ten elements was HQ_ing_ > HQ_derm_ > HQ_inh._ The carcinogenic risks showed that the values of Cd, Ni, Cr, As, and Co were 1.56155E-11, 8.40675E-10, 1.58151E-07, 6.0499E-12, and 4.1299E-09, respectively. Cr had the maximum, followed by Co and As.
3. A multivariate statistical and PMF analysis, as well as Pb and Cu isotopes, showed that PC1 (Cr, Cu, Co, Mn, Ni, Pb, and Zn) primarily comes from natural background and man-made sources; PC2 (Cd) primarily comes from the man-made sources; PC3 (As and Hg) comes from natural background sources. This study can provide references for heavy metals pollution prevention and protection of the environment in the Aibi lake basin and the arid region of northwest China as well as central Asia.

## Figures and Tables

**Table 1:** Reference doses for the three routes of exposure.

Metals	Reference doses ((mg/(kg.d))		
	RfD_ing_	RfD_inh_	RfD_derm_
Cu	0.04	0.012	0.04
Hg	0.0003	0.0003	0.000024
Zn	0.3	0.3	0.06
Pb	0.0035	0.00352	0.000525
Cd	0.001	0.001	0.00001
As	0.0003	0.000123	0.0003
Ni	0.02	0.0206	0.0008
Cr	0.003	0.0000286	0.000075
Co	0.02	0.0000571	0.016
Mn	0.46	0.000014	—

**Table 2:** Exposure amount of (metalloid) heavy metals for children and adults in farmland soil.

	ADI_ing_		ADI_inh_		ADI_derm_	
	Children	Adults	Children	Adults	Children	Adults
Cu	0.000155076	9.59628*E*-06	2.85066*E*-09	2.21561*E*-09	2.48121*E*-07	5.84413*E*-08
Zn	0.000560127	3.46613*E*-05	1.02965*E*-08	8.00269*E*-09	8.96203*E*-07	2.11088*E*-07
Cd	2.7165*E*-08	8.405*E*-09	4.99356*E*-13	1.94057*E*-12	4.3464*E*-11	5.11865*E*-11
Ni	1.11425*E*-05	3.44755*E*-06	2.04825*E*-10	7.95979*E*-10	1.7828*E*-08	2.09956*E*-08
Pb	7.38405*E*-05	4.56934*E*-06	1.35736*E*-09	1.05498*E*-09	1.18145*E*-07	2.78273*E*-08
Cr	4.19233*E*-05	1.29713*E*-05	7.70648*E*-10	2.99485*E*-09	6.70772*E*-08	7.89953*E*-08
As	1.56644*E*-05	4.84665*E*-06	2.87948*E*-10	1.11901*E*-09	2.5063*E*-08	2.95161*E*-08
Hg	3.8231*E*-07	2.36578*E*-08	7.02775*E*-12	5.46216*E*-12	6.11696*E*-10	1.44076*E*-10
Co	5.47385*E*-05	1.45169*E*-06	8.62477*E*-11	3.3517*E*-10	8.75817*E*-08	8.84082*E*-09
Mn	0.000155076	0.000273131	8.1136*E*-08	6.30612*E*-08	7.06208*E*-06	1.66337*E*-06
Total	0.001067998	0.000344707	9.69977*E*-08	7.95869*E*-08	8.52275*E*-06	2.09927*E*-06

**Table 3:** Noncarcinogenic risk (HQ) of each (metalloid) heavy metal and total risks (HI).

	Children				Adults			
	HQ_ing_	HQ_inh_	HQ_derm_	HI	HQ_ing_	HQ_inh_	HQ_derm_	HI
Cu	0.0038769	2.37555*E*-07	6.20303*E*-06	0.00388334	0.000239907	1.84634*E*-07	1.46103*E*-06	0.000241553
Zn	0.00186709	3.43217*E*-08	1.49367*E*-05	1.50054*E*-05	0.000115538	2.66756*E*-08	3.51813*E*-06	0.000119082
Cd	0.000027165	4.99356*E*-10	4.3464*E*-06	4.3474*E*-06	0.000008405	1.94057*E*-09	5.11865*E*-06	1.35256*E*-05
Ni	0.000557125	9.94296*E*-09	0.000022285	2.23049*E*-05	0.000172378	3.86398*E*-08	2.62445*E*-05	0.000198661
Pb	0.021097286	3.85614*E*-07	0.000225038	0.00022581	0.001305526	2.9971*E*-07	5.30044*E*-05	0.00135883
Cr	0.013974433	2.69457*E*-05	0.000894363	0.00094825	0.004323767	0.000104715	0.001053271	0.005481752
As	0.052214667	2.34104*E*-06	8.35433*E*-05	8.82254*E*-05	0.0161555	9.09764*E*-06	0.000098387	0.016262985
Hg	0.001274367	2.34258*E*-08	2.54873*E*-05	2.55342*E*-05	7.88593*E*-05	1.82072*E*-08	6.00317*E*-05	0.000138909
Co	0.002736925	1.51047*E*-06	5.47386*E*-06	8.49479*E*-06	7.25845*E*-05	5.86988*E*-06	5.52551*E*-07	7.90069*E*-05
Mn	0.000337122	0.005795429	—	—	0.000593763	0.004504371	—	0.005098134
Total	0.09796308	0.005826918	0.001281677	0.005221312	0.023066228	0.004624623	0.001301589	0.028992439

**Table 4:** Correction coefficients of ten heavy metals in the soils.

	As	Cr	Cd	Cu	Co	Hg	Mn	Ni	Pb	Zn
As	1									
Cr	0.088^*∗*^	1								
Cd	−0.12^∗∗^	0.202^∗∗^	1							
Cu	0.208^∗∗^	0.583^∗∗^	−0.043	1						
Co	0.003	0.716^∗∗^	0.087^*∗*^	0.772^∗∗^	1					
Hg	−0.038	−0.067	0.117^∗∗^	−0.075	−0.139^∗∗^	1				
Mn	0.058	0.665^∗∗^	0.158^∗∗^	0.675^∗∗^	0.842^∗∗^	−0.089^*∗*^	1			
Ni	−0.023	0.771^∗∗^	0.173^∗∗^	0.623^∗∗^	0.764^∗∗^	−0.066	0.665^∗∗^	1		
Pb	0.194^∗∗^	0.315^∗∗^	−0.188^∗∗^	0.452^∗∗^	0.438^∗∗^	−0.202^∗∗^	0.448^∗∗^	0.221^∗∗^	1	
Zn	0.228^∗∗^	0.524^∗∗^	−0.086^*∗*^	0.767^∗∗^	0.663^∗∗^	−0.127^∗∗^	0.669^∗∗^	0.459^∗∗^	0.663^∗∗^	1

Note: ^*∗*^ indicates significance at *P* < 0.05 level; ^∗∗^ indicates significance at *P* < 0.01 level.

**Table 5:** Contents of dust/soils heavy metals of China and abroad.

Area	As (mg/kg)	Cd (mg/kg)	Cr (mg/kg)	Cu (mg/kg)	Zn (mg/kg)	Pb (mg/kg)	Ni (mg/kg)	Co (mg/kg)	Hg (mg/kg)	Mn (mg/kg)	References
Zhundong, Xinjiang,China, dust	40.40	n.a	482.07	78.66	5840.19	20.10	n.a	n.a	n.a	n.a	Yang et al. [41]
Urumuqi, Xinjiang, China, dust	7.66	n.a	66.07	79.49	648.16	1.4.46	n.a	n.a	n.a	n.a	Yang et al. [41]
Xuzhou, China, soils	n.a	0.41	83.33	26.99	122.11	29.89	26.03	n.a	n.a	n.a	Shan et al. [42]
Tehran, Iran, dust	n.a	10.7	31	203	791	190	31	n.a	n.a	1176.3	Kurt-Karakus, [43]
Selangor, Malaysia, dust	n.a	250	n.a	n.a	210	430	510	n.a	n.a	n.a	Mamat et al. [5]
26 urban atmosphere dust, China	18.605	2.896	80.812	79.544	806.631	115.381	37.318	n.a	71.772	n.a	Mamat et al. [5]
Seven cities, soils, India	23	9.46	377	106.667	214.333	271.667	66	n.a	1.95	n.a	Mamat et al. [5]
72 examined mine soils, China	195.5	11.0	84.28	211.9	1163	641.3	106.6	n.a	3.82	n.a	Li et al. [34]
146 cities, China, soils	12.207	1.497	70.093	44.604	154.203	55.143	41.968	n.a	0.371	n.a	Mamat et al. [5]
Aibi lake, Xinjiang, China, soils	20.011	0.035	53.557	16.981	61.334	8.086	14.235	5.994	0.042	483.311	This reference

n.a: not reported.

## Data Availability

All the data have been included in the paper.
